# Genetic variation in genes involved in folate and drug metabolism in a south Indian population

**DOI:** 10.4103/0971-6866.80359

**Published:** 2011-05

**Authors:** Padmalatha S Rai, T. S Murali, T. G Vasudevan, Shama K. Prasada, Ashok Kumar Bhagavath, Pranita Pai, P. M. Gopinath, K. Satyamoorthy

**Affiliations:** Department of Biotechnology, Manipal Life Sciences Center, Manipal University, Manipal, India

**Keywords:** *MTHFD1*, *TYMS*, *SHMT1*, *MTR*, *MTRR*, *CBS*, *SULT1A1*, polymorphism, south Indian

## Abstract

**BACKGROUND:**

Genetic variations represented as single nucleotide polymorphisms (SNPs) vary across the world population. This genetic polymorphism (such as SNPs) plays an important role in pharmacogenomics. SNPs that affects cellular metabolism, by altering the enzyme activity, have an important role in therapeutic outcome. Allele frequencies in number of clinically relevant SNPs within south Indian populations are not yet known. Hence, we genotyped randomly selected unrelated south Indian subjects from different locations of south India representing the heterogeneous ethnic background of the population.

**MATERIALS AND METHODS:**

Common variants of *MTHFD1, TYMS, SHMT1, MTR, MTRR, CBS and SULT1A1* gene polymorphisms were screened from healthy unrelated south Indian volunteers. Genotypes were determined using RFLP analysis of polymerase chain reaction-amplified products and confirmed by DNA sequencing. Chi-square test was performed to test for deviation from the Hardy-Weinberg equilibrium for each locus.

**RESULTS:**

Gene allele frequency for several polymorphisms in our study differed significantly between the populations of other nations reported for several of the SNPs. These results demonstrate that the populations in different geographic regions may have widely varying genetic allele frequencies for clinically relevant SNPs.

**CONCLUSION:**

The present study reports, for the first time, the frequency distribution of *MTHFD1, TYMS, SHMT1, MTR, MTRR, CBS and SULTIA1* gene polymorphisms in a south Indian population. Population-specific genetic polymorphism studies will help in practicing pharmacogenomic principles in the clinics.

## Introduction

The frequencies of genomic variants vary greatly between the different populations. Sequence variations in humans can affect the development of diseases and response to pathogens, chemicals, drugs, vaccines and other agents. Single nucleotide polymorphisms (SNPs) are also thought to be key enablers in realizing the concept of personalized medicine. These polymorphisms can therefore be used to discern small differences both within a population and among different populations. Heterogeneity in patient response to drug treatment is consistently observed across patient populations. In addition, genetic polymorphisms in drug metabolizing enzymes and other molecules are responsible for much of the interindividual differences in the efficacy and toxicity for chemotherapeutic agents.[1,2] Folate, a vitamin of the B group involved in one-carbon group metabolism, plays an important role in DNA synthesis and methylation. Several polymorphisms in the genes involved in folate uptake and biotransformation have been shown to be associated with the risk of cancer and to anticancer drug response.[3] Folate pathway genes are found to be highly polymorphic and, hence, we studied common polymorphisms in 5, 10-methylene-tetrahydrofolate dehydrogenase1 (*MTHFD1* 1958G>A), methionine synthetase (*MTR* 2756A>G), methionine synthetase reductase (*MTRR* 66A>G), serine hydroxymethyltransferase (*SHMT1* 1420C>T), thymidylate synthase (*TYMS* 2R/3R) and cystathionine-beta-synthase (*CBS* T833C/844INS68) of the folate pathway and the common polymorphism in cytosolic sulfotransferases (*SULT1A1* 638G>A) gene involved in sulfation reaction. MTHFD1 is a trifunctional nicotinamide adenine dinucleotide phosphate (NADP)-dependent cytoplasmic enzyme that catalyzes the conversion of tetrahydrofolate to the corresponding 10-formyl, 5, 10-methenyl and 5,10-methylene derivatives. 10-formyltetrahydrofolate and 5, 10-methylenetetrahydrofolate are the donor cofactors for *de novo* purine and pyrimidine biosynthesis and, thus, the biosynthesis of DNA. *MTHFD1* [5, 10-methylene-tetrahydrofolate dehydrogenase1] gene is located on chromosome 14q23.3 and is 71,654 bases in size. It has a total of 28 exons and codes for a protein that is composed of 935 amino acids. A total of 390 SNPs have been found to be associated with this gene.[4] This study is based on *MTHFD1* G>A polymorphism, where the mutation occurs in exon 21 at nucleotide position 1958.*MTR*[5-methyltetrahydrofolate-homocysteine methyltransferase] encodes the enzyme 5-methyltetrahydrofolate-homocysteine methyltransferase. This enzyme, also known as cobalamin-dependent methionine synthase, catalyzes the final step in methionine biosynthesis. The *MTR* gene is located on chromosome 1q43 and is 105.2 kb in size. It has a total of 33 exons and codes for a protein that is composed of 1265 amino acids. This study is based on *MTR* A>G polymorphism, where the mutation occurs in exon 13 at nucleotide position 2756. The A>G polymorphism at position 2756 in the protein-binding region of *MTR* replaces aspartic acid with glycine.[5] MTRR (5-methionine synthase reductase) is an essential amino acid required for protein synthesis and one-carbon metabolism. Its synthesis is catalyzed by the enzyme methionine synthase. Methionine synthase eventually becomes inactive due to the oxidation of its cob (I) alamin cofactor. The protein encoded by this gene regenerates a functional methionine synthase via reductive methylation. The *MTRR* gene is located on chromosome 5p15.31 and is 320.3 kb in size. It has a total of 15 exons and codes for a protein that is composed of 725 amino acids. The *A66G* polymorphism in the *MTRR* gene results in the substitution of isoleucine with methionine at codon 22.[6] Serine hydroxymethyltransferase (*SHMT*) encodes a vitamin B6-dependent enzyme that catalyzes the reversible conversion of serine and tetrahydrofolate (THF) to glycine and methylene THF. There are two distinct *SHMT* isoenzymes, one in the cytosol localized to the SHMT1 gene on chromosome 17p11.2 and the other in the mitochondrion localized to the *SHMT2* gene on chromosome 12q13.2.9. SHMT1 plays a pivotal role in providing one-carbon units for purine, thymidylate and methionine synthesis, in addition to other metabolic functions. The exact function of SHMT2 is not known.[3] The SHMT1 gene is located on chromosome 17p11.2 and is 35.6 kb in size. It has a total of 12 exons and codes for a protein that is composed of 483 amino acids. The polymorphism of the *SHMT1* C1420T gene results in the substitution of phenylalanine to leucine codon 474. *TYMS* (Thymidylate synthase) catalyzes the methylation of deoxyuridylate to deoxythymidylate using 5, 10-methylenetetrahydrofolate (methylene-THF) as a cofactor. This function maintains the dTMP (thymidine-5-prime monophosphate) pool critical for DNA replication and repair. The *TYMS* enhancer region contains a series of 28-base pair tandem repeats. Two repeats (2 rpt) or three repeats (3 rpt) are most common, with 3 rpt occurring most frequently. More repeats have been observed but are rare.[7,8] The *TYMS* gene is located on chromosome 18p11.32 and is 15,975 bases in size. It codes for a protein that is composed of 313 amino acids. *CBS* (cystathionine-beta-synthase). The protein encoded by this gene acts as a homotetramer to catalyze the conversion of homocysteine to cystathionine, the first step in the trans-sulfuration pathway. The encoded protein is allosterically activated by adenosyl-methionine and uses pyridoxal phosphate as a cofactor. The *CBS* gene is located on chromosome 21q22.3 and is 23,753 bases in size. It codes for 551 amino acids. SULT1A1 is sulfotransferase enzymes that catalyzes the sulfate conjugation of many hormones, neurotransmitters, drugs and xenobiotic compounds.*SULT1A1* gene is located on chromosome 16p12.1 and is 180,005 bases in size, and codes for a protein that is composed of 295 amino acids.*SULT1A1* G638A polymorphism results in G>T transition and in the arginine to histidine replacement. It has been well established that the frequencies of genomic variants can vary greatly between the populations of different countries. We sought to quantify the allele frequencies for *MTHFD1, TYMS, SHMT1, MTR, MTRR, CBS* and *SULT1A1* gene polymorphism in a south Indian population. Further, we compared the allele frequencies for the south Indian population, as a whole, with earlier reported frequencies for different ethnicities.

## Materials and Methods

### Sample collection

Five milliliters of EDTA-anticoagulant blood was collected from healthy unrelated south Indian volunteers for analysis of genotype of *MTHFD1, TYMS, SHMT1, MTR, MTRR, CBS*and *SULT1A1* polymorphism. Informed consent for participation in the study was obtained from all the volunteers, and this study was approved by the ethical committee of Manipal University, Manipal, India. Genomic DNA was extracted from whole blood samples using the phenol-chloroform methodology.

### Genotyping

The *MTHFD1* G1958A (rs2236225), *TYMS* (3R/2R) (rs2236225), *SHMT1* C1420T (rs1979277), *MTR* A2756G (rs1805087), *MTRR* A66G (rs1801394), *CBS* T833/844INS68 and *SULT1A1* G638A (rs1042028) genotypes were analyzed by polymerase chain reaction (PCR)-based RFLP methods and DNA sequence analysis. The PCR primers and restriction enzymes used for the detection of genotypes are given in Table 1. PCR amplification was performed in a thermocycler (Eppendorf, Germany). The primers used for the PCR were purchased from Sigma-Proligo and Sigma Genosys, Bangalore, India. Taq polymerase was purchased from Invitrogen and restriction enzymes were purchased from New England Biolabs, Beverly, MA, USA. DNA sequencing of each of the three genotypes in the *MTHFD1, TYMS, SHMT1, MTR, MTRR, CBS*and *SULT1A1* gene polymorphisms was performed using an automatic DNA sequencer (Applied Biosystems 3130, USA).

**Table 1:** Primers and restriction enzymes used in this study for PCR-RFLP

Gene/polymorphism	Primer sequences 5’S–V3’S	PCR product in bp	Restriction enzyme
*MTHFD1* G1958A (rs 2236225)	5’-CATTCCAATGTCTGCTCCAA-3’	254 bp	Hpa II
	5’-GTTTCCACAGGGCACTCC-3’		
*TYMS* 3R/2R (rs34743033)	5’-GAGCCGGCCACAGGCAT-3’	2R/2R- 294 bp	-
	5’-CGTGGCTCCTGCGTTTCC-3’	3R/3R-322 bp	
*SHMT1* C1420T (rs1979277)	5’-CTG GCA GGG GAT AAG TAC CA-3’ 5’-CCC GCT CCT	108 bp	Ear1
	TTA GAA GTC AG-3’		
*MTR* A2756G (rs1805087)	5’-TGTTCCAGACAGTTAGATGAAAATC-3’	211 bp	HaeIII
	5’-GATCCAAAGCCTTTTACACTCCTC-3’		
*MTRR* A66G (rs1801394)	5’-GCAAAGGCCATCGCAGAAGACAT-3’	66 bp	NdeI
	5’-GTGAAGATCTGCAGAAAATCCATGTA-3’		
*CBS* T833C/844INS68	5’-GTTGTTAACGGCGGTATTGG-3’	w-171 bp m-239 bp	Bsr1
	5’-GTTGTCTGCTCCGTCTGGTT-3’		
*SULT1A1* G638A (rs1042028)	5’-GTTGGCTCTGCAGGGTTTCTAGGA-3’	333 bp	HaeII
	5’-CCCAAACCCCCTGCTGGCCAGCACCC-3’		

w, homozygous wild-type, m, homozygous mutant

### Statistical analysis

Genotype and allele frequencies were calculated under assumption of Hardy-Weinberg equilibrium (HWE). The difference in allele frequencies was determined using Fisher’s exact and χ^2^tests.

## Results

The seven SNPs of folate and drug metabolizing pathway genes, namely *MTHFD1* G1958A (rs2236225), *TYMS*3R/2R (rs2236225), *SHMT1*C1420T (rs1979277), *MTR* A2756G (rs1805087), *MTRR* A66G (rs1801394), *CBS* T833/844INS68 and *SULT1A1* G638A (rs1042028), were analyzed in healthy unrelated south Indian samples [Table 2]. These genes play an important role in pharmacogenomics [Table 3]. Genotype analysis were performed using PCR-RFLP and confirmed using DNA sequencing from DNA isolated from peripheral blood. Genotypes for *MTHFD1, TYMS, CBS* and *SULT1A1* were found to conform to the HWE, whereas those for *SHMT1, MTR and MTRR* were not in HWE. The three SNPs were not in HWE, and the lack of HWE may, however, be due to population stratification, and this was not investigated further. Thus, our results should be interpreted with some caution due to the deviation in HWE [Table 2]. Genotype distributions were as follows: for *MTHFD1* G1958A, 20.8% GG, 48.6% GA, 30.6% 3AA; *TYMS* 3R/2R, 23% 3R/3R, 58% 3R/2R, 19% 2R/2R; *SHMT* C1420T, 97.5% CC, 2.5.% CT, 0%TT; *MTR* A2756G, 67% AA, 32% AG, 1% GG; *MTRR* A66G, 1.7% AA, 62.6% AG, 35.7% GG; *CBS* T833C/844INS68, 97.5% homozygous wild type, 2.5% heterozygous, 0% homozygous mutant; *SULT1A1* G638A, 23% GG, 58% GA, 19% AA. Although a large-scale genotyping of south Indian population has not been undertaken, we compared the allelic frequency obtained from the south Indian population in our study with the available reports for other populations [Table 4]. The south Indian cohort was found to have two SNPs (*TYMS 2R* and *MTR 2756G*) allele frequencies most similar to the Caucasian population [Table 4]. Likewise, the south Indian population was found to be more similar to the Irish population reported for *MTHFD1* 1958A frequency (0.55 and 0.54). It is of interest, however, to note the wide range of frequency variation in *MTRR* 66G with Australian, Chinese and French reports of a frequency of 0.36, 0.26 and 0.14, respectively, whereas the south Indian population of our study showed 0.66 [Table 4].

**Table 2:** Genotype and allele distribution of selected gene polymorphism in a south Indian population, tested for Hardy-Weinberg equilibrium

Gene/polymorphism	Genotype counts (frequency %)			Allele counts (frequency %)		*P*-value	Chi-square value
	w	w/m	m	w	m		
MTHFD1 G1958A (rs 2236225) (n = 150)	31 (20.8%)	73 (48.6)	46 (30.6)	135 (45)	165 (55)	0.84	0.043
TYMS 3R/2R (rs34743033) (n = 94)	21 (22.3%)	54 (57.5)	19 (20.2)	96 (51)	92 (49)	0.15	2.08
SHMT1 C1420T (rs1979277) (n = 100)	15 (15%)	71 (71)	14 (14)	101 (50.5)	99 (49.5)	0.00	27.65
MTR A2756G (rs 1805087) (n = 294)	197 (67%)	94 (32)	03 (1)	488 (83)	100 (17)	0.02	5.17
MTRR A66G (rs 1801394) (n = 294)	05 (1.7%)	184 (62.6)	105 (35.7)	194 (33)	394 (67)	0.00	50.75
CBS T833C/844INS68 (n = 81)	79 (97.5%)	2 (2.5)	0 (0)	160 (99)	2 (1)	0.91	0.013
SULT1A1 G638A (rs 1042028) (n = 128)	30 (23%)	74 (58)	24 (19)	134 (52)	122 (48)	0.07	3.23

w, homozygous wild-type, m, homozygous mutant, w/m, heterozygous mutant. If *P* <0.05, not consistent with Hardy-Weinberg equilibrium (Chi-square test for Hardy-Weinberg equilibrium)

**Table 3:** Drugs associated with the selected genes

Gene	Drugs associated with the gene	Reference
*MTHFD1*	Methotrexate, folic acid	23
*TYMS*	Methotrexate, 5-Flurouracil, Pemetrexed,	24
	Leucovorin, Thymitaq, Doxorubicin,	
	Asparaginase, Cytarabine, Daunorubicin,	
	Etoposide, Irinotecan, Mercaptopurine	
*SHMT1*	Methotrexate, Vitamin B12, Folic Acid	25
*MTR*	Methotrexate, Folic Acid, Vitamin B12, Vitamin	26
	B6, 5-Flurouracil, Pheredoxin, Vertiporfi n	
*MTRR*	Methotrexate, Folic Acid, Vitamin B12, Vitamin	27
	B6, 5-Flurouracil	
*CBS*	Pyridoxine, 5-Flurouracil, 3-Deazaadenosine	28
	ameliorate, 1-b-D-arabinofuranosylcytosine	
	(ara-C), Pravastatin, Folic acid	
*SULT1A1*	Estradiol 17-Beta, Tamoxifen	29

**Table 4:** Frequencies of selected gene alleles reported in different populations of the world

Gene/polymorphism	Population	Number of participants	Allele counts	(frequency)	Reference
					
			m	W	
*MTHFD1* G1958A	Indians	150	0.55	0.45	Present study
(rs 2236225)	Iranian	100	0.52	0.48	9
	Canadian	165	0.58	0.42	10
	Irish	635	0.54	0.46	9
	Chinese	770	0.76	0.24	11
*TYMS* 3R/2R (rs 34743033)	Indians	94	0.49	0.51	(Present study)
	Indian Asian	139	0.64	0.36	12
	Japanese	494	0.15	0.85	13
	Caucasian	114	0.505	0.495	14
	France	202	0.60	0.40	15
*SHMT1* C1420T	Indians	100	0.49	0.51	(Present study)
(rs1979277)	Portuguese	200	0.265	0.735	16
	Caucasian	114	0.38	0.62	14
	France	205	0.33	0.673	15
*MTR* A2756G (rs 1805087)	Indians	294	0.17	0.83	Present study
	Portuguese	200	0.167	0.849	16
	Chinese	220	0.08	0.92	17
	Caucasian	114	0.17	0.83	14
*MTRR* A66G (rs 1801394)	Indians	294	0.67	0.33	Present study
	Australians	248	0.36	0.64	18
	Chinese	220	0.26	0.74	17
	France	206	0.14	0.76	15
*CBS* T833C/844INS68	Indians	81	0.01	0.99	Present study
	Czech	200	0.07	0.93	19
	Africans	190	0.33	0.77	19
	British	82	0.10	0.90	20
*SULT1A* (rs 1042028)	Indians	100	0.48	0.52	Present study
	Chinese	290	0.080	0.91	21
	Caucasian	245	0.332	0.656	21
	African-American	70	0.294	0.477	21

## Discussion

Our study was designed to analyze the genetic polymorphisms of six SNPs, namely *MTHFD1,* G1958A, *TYMS* 3R/2R, *SHMT1* C11420T, *MTR* A2756G, *MTRR* A66G and *SULT1A1* G638A polymorphism in randomly selected unrelated south Indian subjects from different locations in this region of the country representing the heterogeneous ethnic background of the population. To date, no study has been carried out in the south Indian population to determine the frequencies of the *MTHFD1, TYMS, SHMT1, MTR, MTRR* and *SULT1A1* alleles that are important in pharmacogenomics. This study assessed SNPs from genes involved in the biological activity of drug metabolism, DNA synthesis and DNA methlylation.[3] *MTHFD1* allelic frequencies were found to be similar to Irish and Canadian populations, while it varied significantly with the Chinese population.[9–11] We found that the allelic frequencies in the south Indian population are similar to the Caucasians for two SNPs (*TYMS* and *MTR*),[14] but they differ significantly from the other populations.[12–17] *MTRR, CBS* and *SULT1A1* differ significantly from the other populations.[15,17,18–21] Our recent reports on *TPMT* gene polymorphism from the same population also showed a significant difference in allelic frequency with other populations.[22] These results indicate that genotype data from one group or subgroup (i.e., nation or ethnicity) should not be overly generalized and applied to genetically distinct groups (i.e., other nations or ethnicities).[1] These observations will have a significant impact in understanding the therapeutic response to various drugs[23–29] In conclusion, our study confirmed that great genomic diversity exists among different ethnicities. Our results should be interpreted with some caution, due to the deviation of *SHMT1, MTR* and *MTRR* alleles in HWE. The findings need careful interpretation and confirmation in studies involving a larger sample size. In a country like India, where we have a mixture of races and a large socio-economic variation, there is a need for initiative in this field to provide the best medical care to all individuals.
